# Effectiveness of case based cultural competency among nurses working in private hospitals of Bangkok, Thailand: A Quasi-experimental study

**DOI:** 10.12669/pjms.341.14080

**Published:** 2018

**Authors:** Pimkhwan Bunjitpimol, Ramesh Kumar, Ratana Somrongthong

**Affiliations:** 1 Dr. Pimkhwan Bunjitpimol, PhD. College of Public Health Sciences, Chulalongkorn University, Bangkok, Thailand; 2 Dr. Ramesh Kumar, MBBS, MSPH, PhD. Health system and Policy Department, Health Services Academy, Islamabad, Pakistan; 3 Dr. Ratana Somrongthong, PhD. Associate Professor, College of Public Health Sciences, Chulalongkorn University, Bangkok, Thailand

**Keywords:** Effectiveness, Intervention, Nursing competence, Private hospital, Work environment

## Abstract

**Objectives:**

To evaluate the effect of case based cultural competency level and its factors affecting on nurse job in two private hospitals of Bangkok, Thailand.

**Methods:**

This was a quasi-experimental study implemented the cased-based cultural competent intervention in two private hospitals of Thailand in 2015. One hundred sixty six nurses from two control and intervention private hospitals through simple random selection method were selected for this study. Data was collected at the beginning of study (pre-test), immediately after the intervention and after two months of intervention (post-test). Tool was pretested, validated and piloted before to conduct study.

**Results:**

Total 166 nurses were included in this study. The characteristics among the study participants were similar between both hospitals at the baseline and found statistically non-significant (p= > 0.05). However, after the intervention the cultural knowledge, attitude and practice competency score levels have significantly increased among the nurses in the intervention group as compared to control group (p= < 0.05).

**Conclusion:**

Study has concluded that an intervention has positively affected on cultural knowledge, practice and attitude competency among nurses working in private hospital of Bangkok Thailand.

## INTRODUCTION

Globally health is always remained a basic need of general masses. However, the healthcare cost is more expensive with long waiting queue for patient in UK, Japan, USA, Canada, Middle East and Europe. Developing countries like; Brazil, Bolivia, Costa Rica, Hungary, Poland, Belgium India, Cuba, Jordan, Lithuania, Malaysia, Israel, Singapore and South Korea have lesser waiting queue with low cost of treatment as compare to developed countries. Medical tourism is more popular in Thailand during last decade; this would indirectly benefiting the economy of country and also sharing their culture with foreigners.^1^ Data shows that majority of patients visiting this country are coming from Japan, United States, Europe, and the Middle East and their average expenses were calculated as US$1,000 per visit.^2^

In addition to Thailand, the Patients are also visiting the countries like India, Singapore, and Malaysia for their health consultations. However, Thailand should focus more towards cultural training culturally sensitive and care for the healthcare workers in international hospitals by promoting the knowledge, attitude and practices among their medical staff.^3^-^6^ Nurse should be trained to communicate in international language in fluent way so that patients can understand them easily and respond.^7^ Studies have proved that healthcare workers exposure regarding cultural competence is very imperative in motivating the patients in organization.^8^

There is intense need to care the patient as per their cultural practices and belief within organization but most international hospitals does not have that kind of abilities and available trained staff to handle international patients as per their need. Most essential skill is communication and handling of patient should be adopted in all institutions prior to start the international healthcare for patients.^7^ However, in most of the health organization nurses are the front players in health to handle and mange the patients with in any hospital.^9^ They should be trained during their training on multi-cultural care of patients as an independent training.^10^

By keeping in view the above discussed situation, this particular intervention study was conducted to accomplish this urgent need and also focus the hospital mangers towards this neglected issue. This study also aims to highlight the cultural trainings gap with non-governmental hospitals and to evaluate the effectiveness of this research model to achieve better results within the hospitals to promote medical tourism.

## METHODS

Quasi-experimental study design was adopted during this study by selecting one hospital as a control and other as intervention located in Bangkok city of Thailand. The samples were selected using simple random sampling (SRS) method (n=166), 83 nurses from each hospital working in different departments. The study aimed to explore a minimum of 40% of total nurses working in both hospitals out of 350. Nurses working on regular basis since one year were eligible for the study and pregnant nurses were excluded. Review board of College of Public Health Science, Chulalongkorn University (COA No.186/2557) has ethically approved this study. Written consent was taken prior to conduct the study from the respondents and administration approval was taken from both concerned hospitals.

A self-administered questionnaire was modified and used to gather data about age, gender, marital status, religion, education, position, responsibility, work experience, salary, work shift, language skills and feeling and confidence in communication and nursing care towards foreign patients and cultural competency. The questionnaire consisted of 4 parts like; demographic and experience factors (15 questions), knowledge of different culture (116 knowledge questions in food, greeting, religions, belief, dress code, language, manner, healthcare, tradition and social), and attitude towards cultural competency (14 questions): and practice of cultural competent nursing care (10 questions). Pre-test of questionnaire was done on 33 nurses in another private hospital with similar kind of facilities to check the reliability of tool and content validity were checked by five experts in relevant field. Bloom's criteria were used to classify level of knowledge.^11^ Mean score was used to know the level of knowledge, attitude and practices as per Bloom's criteria. The study involved implementation of 3-month weekly case-based intervention during the first 3 months of the study and 2-time monthly boosters at the end of month 4 and month 5. Each intervention session was comprised of 3 hours duration. The content of intervention program was the simulation across cultural environment and to support nurses to learn the social skills and cultural competency required for daily life with medical terminology. This programme provided nurses with:

- How to work with and manage foreign patients effectively
- Understanding of different cultures and how it impacts the workplace
- Techniques for motivating with foreign patients
- Strategies to cope and manage with cross cultural issues

The data collection was conducted at the beginning of study (pre-test), after the intervention and after the two months (post-test). Mean, percentage and frequencies were generated for descriptive data and t test was used for continuous variables and the chi square test was used for categorical variables to see the differences in two groups both at baseline and after the interventions. Analysis of covariance was used to test the main and interaction effects of categorical variables on a continuous dependent variable, controlling for the effects of selected other continuous variables, which co-vary with the dependent variables.

## RESULTS

The demographic characteristics and work-related factors of the samples show that the age and level of seniority was found statistically significant difference in control and intervention hospitals. However, sex, marital status, religion, highest education, monthly salary and current responsibilities were found statistically non significant in both hospital at baseline. The work related experiences among respondents in both hospitals were found similar at baseline. However, those nurses working in shifts and with Yawee language were found to have significant difference (Table-I).

**Table-I:** Demographic characteristics and work related factors among nurses.

Variables		Hospital B (Control)	Hospital A (Intervention)	Total	Chi-square Control vs Intervention

		N = 83 (%)	N = 83 (%)	N = 166 (%)	p-value^1^
Sex	Male	2 (2.4%)	2 (2.4%)	4 (2.4%)	1.000
	Female	81 (97.6%)	81 (97.6%)	162 (97.6%)	
Age (years)				
Range = 22-59	22-30	30 (36.1%)	51 (61.4%)	81 (48.8%)	0.003**
(*x̄*±SD = 33.0±8.7)	31-40	29 (34.9%)	21 (25.3%)	50 (30.1%)	
More than 40	24 (28.9%)	11 (13.3%)	35 (21.1%)	
Marital status^3^	Single	46 (55.4%)	51 (61.4%)	97 (58.4%)	0.586
	Married	33 (39.8%)	30 (36.1%)	63 (38.0%)	
	Divorced/widowed	4 (4.8%)	2 (2.4%)	6 (3.6%)	
>Religion	Buddhism	77 (92.8%)	81 (97.6%)	158 (95.2%)	0.277
	Christian/Muslim	6 (7.2%)	2 (2.4%)	8 (4.8%)	
Highest education	Bachelor degree	77 (92.8%)	76 (91.6%)	153 (92.2%)	1.000
	Master degree	6 (7.2%)	7 (8.4%)	13 (7.8%)	
Level of seniority^3^	Junior nurse	0	9 (10.8%)	9 (5.4%)	0.000**
	Nurse	45 (54.2%)	59 (71.1%)	104 (62.7%)	
	Senior nurse	5 (6.0%)	2 (2.4%)	7 (4.2%)	
	Supervisor	29 (34.9%)	10 (12.0%)	39 (23.5%)	
	Others	4 (3.8%)	3 (3.6%)	7 (4.2%)	
Current monthly salary (Baht) (32-34 THB = 1 USD at time of study)^3^	15,000-20,000	3 (3.6%)	9 (10.8%)	12 (7.2%)	0.137
	20,001-30,000	17 (20.5%)	26 (31.3%)	43 (25.9%)	
	30,001-40,000	37 (44.6%)	27 (32.5%)	64 (38.6%)	
	40,001-50,000	20 (24.1%)	16 (19.3%)	36 (21.7%)	
	More than 50,001	6 (7.2%)	5 (6.0%)	11 (6.6%)	
Current Responsible areas (last three months)	Out-patient Department	16 (19.3%)	14 (16.9%)	30 (18.1%)	0.998
	In-patient Department	41 (50.6%)	41 (50.6%)	82 (50.6%)	
	Emergency room	8 (9.6%)	9 (10.8%)	17 (10.2%)	
	Operation room	9 (10.8%0	9 (10.8%)	18 (10.8%)	
	ICU	8 (9.6%)	9 (10.8%)	17 (10.2%)	
Experience as nurse (years)					
Range = 1-39 years	1-3	17 (20.5%)	27 (32.5%)	44 (26.5%)	0.152
(*x̄*±SD = 9.9±8.6)	4-10	32 (38.6%)	33 (39.8%)	65 (39.2%)	
	11-20	21 (25.3%)	17 (20.5%)	38 (22.9%)	
	More than 20	13 (15.7%)	6 (7.2%)	19 (11.4%)
Work shift	Day shift	52 (62.7%)	25 (30.1%)	77 (46.4%)	0.000**
(last three months)	Night shift	31 (37.3%)	58 (69.9%)	89 (53.6%)	
Experience with foreign patients	No	24 (28.9%)	20 (24.1%)	44 (26.5%)	0.598
	Yes	59 (71.1%)	63 (75.9%)	122 (73.5%)	
Language skills (English)	Poor	10 (12.0%)	15 (18.1%)	25 (15.1%)	0.516
	Moderate	64 (77.1%)	61 (73.5%)	125 (75.3%)	
	Good	9 (10.8%)	7 (8.4%)	16 (9.6%)	
Chinese^2^	Poor	81 (97.6%)	75 (90.4%)	156 (94.0%)	0.099
	Moderate/Good	2 (2.4%)	8 (9.6%)	10 (6.0%)	
Yawee^2^	Poor	82 (98.8%)	78 (94.0%)	160 (96.4%)	0.047*
	Moderate/Good	1 (1.2%)	5 (6.0%)	6 (3.6%)	

1 p-value was calculated using a Chi-square test using 2-sided method (accepted level is 0.05)

2 Data was regrouped for categories with cell/cells having expected count of 0 for Chi-square test

3 Fisher's exact test was used for categories with cell/cells having expected count less than 5

* The samples from the two groups are significantly different (p-value < 0.05)

** The samples from the two groups are highly significantly different (p-value < 0.01)

Considering the effects of the demographic characteristics and work-related factors on the levels of the cultural knowledge competency between the two groups, the levels of the cultural knowledge competency of the two groups were significantly different where the knowledge competency of the control group was significantly lower (Table-II).

**Table-II:** The effects of different demographic characteristics & work-related factors on the cultural knowledge competency in nurses from the control and intervention groups using General Linear Model repeated-measures ANCOVA.

Parameter	Type-III Sum of Squares	df	Mean Square	F	p-value
Age	345.582	1	345.582	3.987	0.048*
Level of seniority	389.230	1	389.230	4.491	0.036*
Work shift	178.375	1	178.375	2.058	0.154
Confidence	248.734	1	248.734	2.870	0.092
Yawee language skill	193.472	1	193.472	2.232	0.137

* The data at pre-test is significantly different (p-value < 0.05).

**Fig.1:**
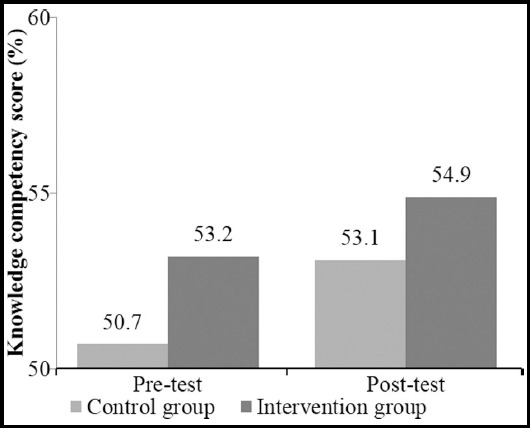
Comparison of the competency score of cultural knowledge between the control and intervention groups at pre-test and post-test.

Taking into account the effect of age and level of seniority, the levels of knowledge competency between two groups were different where knowledge competency of the intervention group was significantly higher than those from the control group (Fig.1).

Taking into account the effect of age and level of seniority obtained from Table-II, the levels of some sub-categories of knowledge competency between two groups were significantly different; these included cultural knowledge related to food, manner and social skills (p-value < 0.05) (Table-III).

**Table-III:** Comparison of the competency score of sub-cultural knowledge between the control and intervention groups at post-test using General Linear Model repeated-measures ANCOVA.

Category	ANCOVA	

	Mean difference	p-value^1^
Food	-4.180	<0.001**
Greeting	-0.180	0.405
Religion/belief	2.400	0.017*
Language/costume	0.000	0.985
Manner	-1.540	<0.001**
Health	-0.020	0.943
Culture	1.780	<0.001**
Social	-5.740	<0.001**

1 p-value was calculated using an ANCOVA test at a significant level of 0.05 (between groups only)

* The samples from the two groups are significantly different (p-value < 0.05)

** The samples from the two groups are highly significantly different (p-value < 0.01).

**Fig.2:**
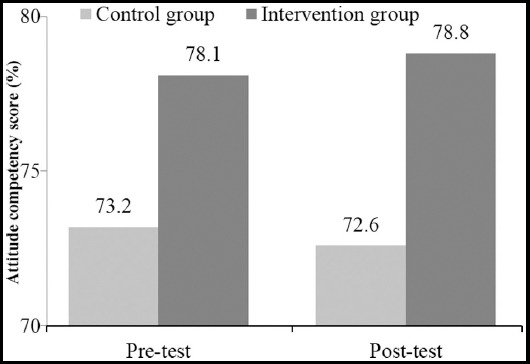
Comparison of the competency score of cultural attitude between the control and intervention groups at pre-test and post-test.

The levels of cultural attitude competency between two groups were significantly different where the competency of intervention group is significantly higher than the control group (Fig.2).

The comparison of the competency score of cultural nursing practice between the control and intervention groups at post-test is shown in Table-IV. It was found that the cultural nursing practice competency score of the nurses from the intervention group was significantly higher than those from the control group (p-value < 0.05).

## DISCUSSION

Successful cross-cultural nursing care is proved to be very helpful for patient care within any organization. Present study results were supported by many researches across the world.^5^,^12^-^14^ Further observation revealed that the levels of cultural knowledge competency and cultural attitude competency of the nurses from the intervention group were significantly higher than those from the control group. This suggests that nurses with different demographic characteristics and work-related factors had different levels cultural competency.^15^

It has been found that the case based intervention package to improve the cultural competency of the nurses in 3 main different categories, namely, knowledge, attitude and nursing practice did not effectively improve the cultural competency levels in nurses. In other words, the knowledge competency was only improved for a short period of time after the 12-weekly cultural competency training and it then subsequently dropped after the 2-monthly cultural competency. This suggested that the nurses could not incorporate or integrate the knowledge into practice where their knowledge faded away over time.

Additionally, the cultural attitude competency was absolutely not affected by the intervention. Also, the intervention package did not have any effect on the cultural competency level in nursing practice. Further, while the overall cultural knowledge competency level was insignificantly affected by the intervention package, the different sub-categories of the cultural knowledge competency were affected differently. Only the knowledge related to food and social practices improved after receiving the intervention package while other sub-categories of the cultural knowledge competency either remained constant or decreased. While there has been number of studies trying to improve the cultural competency in nurses as it has been regarded as an important factor affecting the nursing cares, there has been only few studies conducted in Thai landscape.^16^-^18^

Furthermore, there has only been a case-based study on improving cultural competency of healthcare personals at a nursing school level and reported that the nursing students who had experiences with patients from different cultural backgrounds had significantly higher cultural competency that those with no experiences. This suggested that the cultural knowledge, cultural awareness, cultural skills, willingness to provide cares to patients from different cultures and interaction with patients from different cultures could be improved by gaining experiences with people with different cultures.^18^

Individuals with different behaviors and attitude could be improved through adopting the cultural practices.^15^ This agrees well with the previous study that confidence is one of the factors influencing professional cultural practice as well as career satisfaction and career advancement.^19^ The level of cultural confidence of a person is significantly related to the cultural sensitivity; over-confidence or under-confidence of a person may introduce cultural pain in a person.^20^,^21^

## CONCLUSION

The study shows that cultural competency in nurses were considered as low to moderate. As cultural competency has been identified as an essential component of nursing practice where culturally competent nurses can result in better cares for patients and in turns better healthcare outcomes. The study shows that while knowledge competency level can increase and decrease in a short period of time, the levels of practice and attitude competency may need a longer duration in order to improve.

### Author`s Contribution

**PB** conceived, designed and did statistical analysis & data collection.

**RK** did manuscript editing and writing.

**RS** did review and final approval of manuscript.
